# Cushioned-Density Gradient Ultracentrifugation (C-DGUC) improves the isolation efficiency of extracellular vesicles

**DOI:** 10.1371/journal.pone.0215324

**Published:** 2019-04-11

**Authors:** Phat Duong, Allen Chung, Laura Bouchareychas, Robert L. Raffai

**Affiliations:** 1 Surgical Service San Francisco VA Medical Center, San Francisco, California, United States of America; 2 Division of Vascular and Endovascular Surgery, Department of Surgery, University of California, San Francisco, California, United States of America; Politechnika Slaska, POLAND

## Abstract

Ultracentrifugation (UC) is recognized as a robust approach for the isolation of extracellular vesicles (EVs). However, recent studies have highlighted limitations of UC including low recovery efficiencies and aggregation of EVs that could impact downstream functional analyses. We tested the benefit of using a liquid cushion of iodixanol during UC to address such shortcomings. In this study, we compared the yield and purity of EVs isolated from J774A.1 macrophage conditioned media by conventional UC and cushioned-UC (C-UC). We extended our study to include two other common EV isolation approaches: ultrafiltration (UF) and polyethylene glycol (PEG) sedimentation. After concentrating EVs using these four methods, the concentrates underwent further purification by using OptiPrep density gradient ultracentrifugation (DGUC). Our data show that C-DGUC provides a two-fold improvement in EV recovery over conventional UC-DGUC. We also found that UF-DGUC retained ten-fold more protein while PEG-DGUC achieved similar performance in nanoparticle and protein recovery compared to C-DGUC. Regarding purity as assessed by nanoparticle to protein ratio, our data show that EVs isolated by UC-DGUC achieved the highest purity while C-DGUC and PEG-DGUC led to similarly pure preparations. Collectively, we demonstrate that the use of a high-density iodixanol cushion during the initial concentration step improves the yield of EVs derived from cell culture media compared to conventional UC. This enhanced yield without substantial retention of protein contaminants and without exposure to forces causing aggregation offers new opportunities for the isolation of EVs that can subsequently be used for functional studies.

## Introduction

Extracellular vesicles (EVs), including those referred to as exosomes, are membrane-enclosed microparticles abundantly present in body fluids and are thought to be secreted by all cell types [1]. Recent observations of RNA [2] and metabolite [3] exchange via EVs have led to a boom into their research [4, 5]. Although the exact nature of their biogenesis and function remains incompletely understood, EVs are recognized as intercellular messengers in health and disease [4]. Moreover, EVs are potentially an ideal source of diagnostic biomarkers and delivery vehicles for therapeutic applications [6, 7]. Although there are growing interests in EV biology, progress in this field is hampered by variability and inconsistencies in reports of their function [8]. The source of such biological noise has been proposed to include from their mode of isolation [8, 9]. There is thus a need for the development of new methodologies that can generate highly pure, intact EVs to improve rigor and reproducibility of experiments amongst different laboratories [5, 10–12].

Density gradient ultracentrifugation (DGUC) has long been recognized as a powerful method to reproducibly separate and purify nano-sized biological entities including cellular organelles [13], viruses [14], macromolecules [15], and lipoproteins [16] from complex matrices such as cell homogenates and blood plasma. The use of DGUC has contributed substantially to the discovery of subcellular structures and the elucidation of fundamental processes of cell biology including membrane compartmentalization [17] and lipoprotein metabolism [16]. The practice of DGUC has recently regained popularity as it has proven to be a robust approach for EV purification [10, 18]. This technique is recognized to provide superior quality EV preparations suitable for reliable functional and structural analyses over alternative approaches [19, 20]. Unfortunately, the use of DGUC for EV research has been daunting due in part to the limit of the sample volume that can be processed through ultracentrifugation [9]. DGUC is typically performed using a small amount of sample that is layered on top or below a density gradient. Therefore, it is not readily amenable for EV isolation from large volumes of biofluids and conditioned media. As such, a concentration step is often required before DGUC. The most commonly used concentration approach, ultracentrifugation (UC), has been reported to suffer from a weak recovery of EVs likely due to incomplete sedimentation [21], physical disruption and aggregations during pelleting [22–24]. Such morphological alteration could lead to artifacts and unwanted downstream signaling outcomes [24]. Despite such limitations, recent findings from a worldwide survey of ISEV members indicate that UC remains by far the most commonly used method accounting for 81% of EV isolation [25].

In addition to ultracentrifugation-based approaches, other commonly used isolation approaches to produce concentrated EVs include ultrafiltration (UF) and polyethylene glycol (PEG) sedimentation. Although both of these methods have been reported to recover more EVs than UC, they have also been noted to retain substantial contaminants that could contribute variable signaling affecting reproducibility in studies of EV properties [9, 26].

In an attempt to enhance EV recovery by ultracentrifugation, we built on prior reports [27–29] and developed an approach to concentrate EVs onto a high-density cushion of iodixanol. This method termed C-DGUC avoids harsh conditions associated with direct pelleting [30]. However, the benefits of C-DGUC for EV isolation have so far not been reported. In this study, we sought to determine the value of C-DGUC over other commonly used approaches for EV isolation.

## Materials & methods

### Cell culture

The J774A.1 murine macrophage cell line (ATCC TIB-67) was cultured in Dulbecco’s Modified Eagle’s Medium (DMEM) (Corning) supplemented with 10% fetal bovine serum (Gibco), 1% GlutaMax (Gibco), and 1% penicillin-streptomycin (Gibco). The cells were plated onto 150 mm round dishes at a density of 5 x 10^6^ cells per dish. After the cells reached 80% confluency, they were washed with PBS and cultured in EV-free media (EFM) for 24 hours. Subsequently, media from eight 150 mm dishes containing a total of 2 x 10^8^ cells were pooled to generate 160 mL of culture media. Cultures were prepared on more than three separate occasions.

### Generation of EV-free media

DMEM with 20% fetal bovine serum, 2% GlutaMax, and 2% penicillin-streptomycin was loaded into an ultracentrifuge tube and spun at 100,000 x g in a type 45 Ti rotor overnight. All centrifugation steps in this study occurred at 4°C. The supernatant was collected and filtered through a polyethersulfone filter with a pore size of 0.2 μm. The filtered media was diluted with an equal volume of DMEM.

### Clarification of cell culture media

The J774A.1 culture media was initially spun at 400 x g for 10 minutes and 2,000 x g for 20 minutes. The supernatant was collected and passed through a 0.2 μm polyethersulfone filter. This clarified media is henceforth referred to as the conditioned media (CM). Aliquots of 36 mL of CM were concentrated using one of the following four methods: ultracentrifugation, cushioned-ultracentrifugation, ultrafiltration, and polyethylene glycol precipitation.

### Ultracentrifugation (UC)

An aliquot of 36 mL of CM was transferred to an ultracentrifuge tube and spun at 100,000 x g for 3 hours in a type 50.2 Ti rotor. The supernatant was carefully removed, and the pellet was resuspended in 1 mL of DMEM. To produce a concentrate that contained 40% iodixanol, 2 mL of Optiprep solution containing 60% iodixanol (Sigma-Aldrich), was added to the resuspended pellet to create a 3 mL UC concentrate.

### Cushioned-ultracentrifugation (C-UC)

An aliquot of 36 mL of CM was transferred to an ultracentrifuge tube and underlaid with 2 mL of 60% iodixanol. The tube was spun at 100,000 x g for 3 hours in a type 50.2 Ti rotor. A blunt point needle aspirated the bottom 2 mL of iodixanol and 1 mL of supernatant above the cushion to produce a 3 mL C-UC concentrate.

### Ultrafiltration (UF)

An aliquot of 36 mL of CM was concentrated to a volume of 1 mL using an Amicon Ultrafilter device composed of regenerated cellulose with a 100 kDa molecular weight cut-off (MWCO) as per the manufacturer’s instructions. A 2 mL volume of 60% iodixanol solution was added to produce a 3 mL UF concentrate.

### Polyethylene glycol (PEG) precipitation

Precipitation of EVs using PEG was conducted by taking 9 mL of a solution containing 50% PEG 6000 and 375 mM NaCl and adding it to 36 mL of CM, subsequently this mixture was incubated at 4°C for 18 hours. The mixture was centrifuged at 1500 x g for 30 min. After carefully removing the supernatant, the pellet was resuspended in 1 mL of DMEM. Subsequently, 2 mL of 60% iodixanol solution was added to produce a 3-mL PEG concentrate.

### Density gradient ultracentrifugation (DGUC)

Solutions containing 5%, 10%, 20% iodixanol were prepared by using 60% iodixanol and homogenization buffer composed of 0.25 M sucrose, 1 mM EDTA, and 10 mM Tris-HCl, pH 7.4. A step gradient was produced by first placing 3 mL of the 5% iodixanol solution in the bottom of the tube. Subsequently, 3 mL of the 10% iodixanol was carefully placed below the 5% iodixanol solution, followed by 3 mL of the 20% iodixanol. Concentrates from each of the four different methods were individually placed below the discontinuous gradient. The gradient was then spun at 100,000 x g for 18 hours using an SW 40 Ti rotor. After centrifugation, twelve 1 mL fractions were collected starting from the top of the tube.

### Density measurement

An RBD-6000 refractometer (Laxco) was used to measure the refractive index of each fraction collected from the DGUC gradient. The refractive index was converted to density based on a standard curve of 10, 20, 40, and 60% iodixanol. Each fraction was measured three times from three independent experiments.

### Nanoparticle tracking analysis (NTA)

Nanoparticle tracking analysis was performed on an LM14 Nanosight instrument (Malvern). Samples were typically diluted between 1:50 to 1:400 in PBS to achieve a concentration range of 10^8^−10^9^ nanoparticles per mL. Data were collected as a mean reading of three videos of one minute in length with parameters being set at a camera level of 13 and detection threshold of 3. All NTA measurements were performed in triplicates from three independent experiments.

### Protein quantification

Protein quantification was conducted using a Qubit 3.0 Fluorometer as per the manufacturer’s instructions (Thermo Fisher). All samples were measured once and the average readings of three biological replicates are presented.

### Protein electrophoresis and detection by Coomassie staining

Coomassie staining was performed by taking 90 μL from each fraction after DGUC. Each fraction was mixed with 10 μL of 10x RIPA buffer containing the protease inhibitor phenylmethylsulfonyl fluoride. Subsequently, the fractions were combined with 4 x Laemmli buffer containing 2-Mercaptoethanol. The fractions were boiled at 95°C for 5 minutes, resolved by SDS-PAGE composed of a 3%–15% gradient gel that was ran at 50V overnight. The gel was stained with a solution of Coomassie Brilliant Blue for 2 hours. Subsequently, the gels were de-stained with a solution containing 40% methanol and 10% acetic acid and imaged using an ImageQuant LAS 4000.

### Transmission electron microscopy

Electron microscopy of the samples was conducted by loading 7 x 10^8^ nanoparticles onto a glow discharged 400 mesh Formvar-coated copper grid (Electron Microscopy Sciences). The nanoparticles were left to settle for two minutes, and the grids were washed four times with 1% Uranyl acetate. Excess Uranyl acetate was blotted off with filter paper. Grids were then allowed to dry and subsequently imaged at 120kV using a Tecnai 12 Transmission Electron Microscope (FEI).

### Western blots

Equal volumes or equal nanoparticle numbers were mixed with 4 x Laemmli buffer containing 2-Mercaptoethanol and boiled at 95 °C for 5 minutes. Samples were resolved on a 10% SDS-PAGE gel and transferred onto PVDF using a standard tank transfer protocol. The membranes were blocked with 5% non-fat milk dissolved in PBS for one hour and incubated with one of the following primary antibodies (dilution, company, catalogue number): anti-CD9 (1:500, Abcam, ab92726), anti-CD81 (1:500, Santa Cruz Biotechnology, sc-166029), anti-Alix (1:200, Santa Cruz Biotechnology, sc-53540), anti-Calnexin (1:500, Abcam, ab10286), anti-GM130 (1:250, BD Biosciences, 610823). The primary antibodies were diluted in 1% non-fat dry milk in PBS and probed overnight at 4 °C. The membranes were washed 4 times for 5 minutes at room temperature with PBS containing 0.1% Tween (PBST) and incubated with either anti-Mouse IgG-HRP (1:1000, Santa Cruz Biotechnology, sc-516102) or anti-Rabbit IgG-HRP (1:1000, Thermo Fisher, A16023) diluted in 1% non-fat milk in PBS for 1 hour. The membranes were washed 4 times for 5 minutes with PBST and rinsed twice with PBS before detection. Amersham ECL Prime substrate was used for detection. Each western blot presented is a representative image of three separate biological replicates.

### RNA extraction and qRT-PCR

Total RNA was extracted using the miRNeasy kit (Qiagen) as per the manufacturer’s instructions. Before the RNA isolation, a mixture of RNA synthetic spike-in templates (Qiagen) composed of UniSp2, UniSp4, UniSp5, and cel-miR-39-3p was added to the lysis buffer for normalization purposes. An equal volume of isolated RNA was converted to cDNA using the miRCURY LNA Universal RT microRNA PCR (Qiagen). The qRT-PCR was conducted using the miRCURY LNA SYBR Green PCR Kit (Qiagen) on a QuantStudio 7 Flex Real-Time PCR System. The expression of miR-21 (Qiagen, Cat# YP00206038), miR-146a (Qiagen, Cat# YP00204688), and miR-16 (Qiagen, Cat# YP00205702) were quantified relative to the expression of the RNA spike-in UniSp2 (Qiagen, Cat# YP00203950).

### Statistical methods

All statistical analyses were performed using GraphPad Prism 8.0 (GraphPad Software). Data were analyzed for statistical significance by 1-way ANOVA followed with Dunnett’s multiple comparison test, C-UC served as the control group. A value of P<0.05 was considered significant.

## Results

### Nanoparticle and protein levels following the concentration of conditioned media

**Fig 1:**
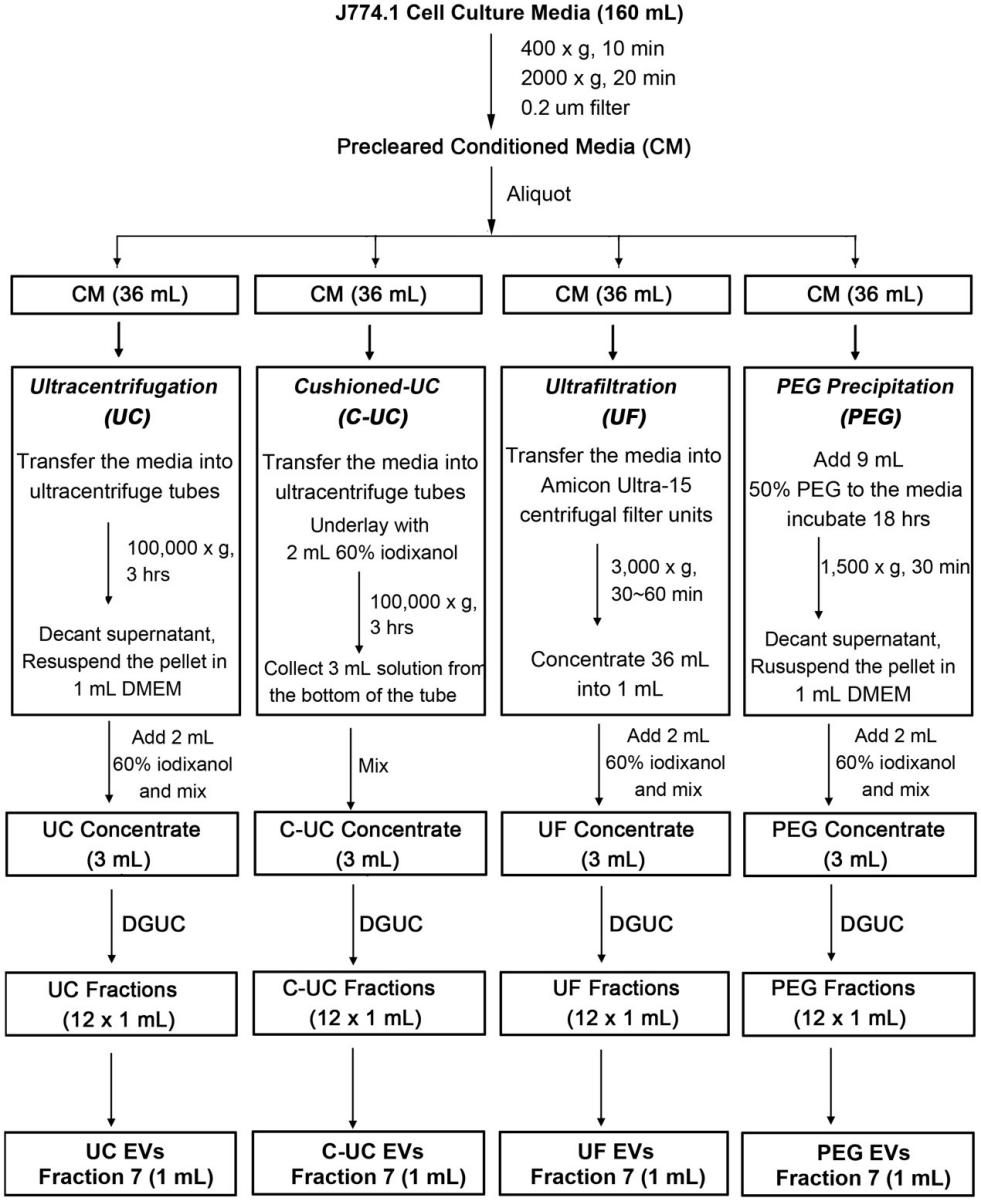
Schematic diagram of the experimental procedure. The J774A.1 murine macrophage cell line was cultured in EV-free media (EFM) for 24 hours. The cell culture media was then collected and subjected to low-speed centrifugation and filtration to clarify cellular debris and large microvesicles. Aliquots of 36 mL of such pre-cleared conditioned media (CM) were concentrated using UC, C-UC, UF or PEG into 1 mL suspensions, which were then mixed with 2 mL 60% iodixanol individually to generate 3 mL concentrates. DGUC then separated the concentrates into twelve 1 mL fractions. The EV-containing fraction, fraction 7 was subsequently used for further analysis.

Cultures of J774A.1 macrophages were grown to 80% confluency at which point the cells were washed with PBS and incubated in complete media that had been depleted of serum EVs. The cells were incubated in such EV-free media (EFM) for 24 hours, and a total volume of 160 mL of cell culture media was collected. As detailed in Fig 1, the media was first cleared of cellular debris by low-speed centrifugation at 400 x g for 10 min and 2000 x g for 20 min. Afterwards, the media was further cleared by filtration through a 0.2 μm membrane to remove larger microvesicles. The conditioned media (CM) was divided into four equal volumes of 36 mL, which were then concentrated with one of the following methods: ultracentrifugation (UC), cushioned ultracentrifugation (C-UC), ultrafiltration (UF) or PEG precipitation (PEG).

**Fig 2:**
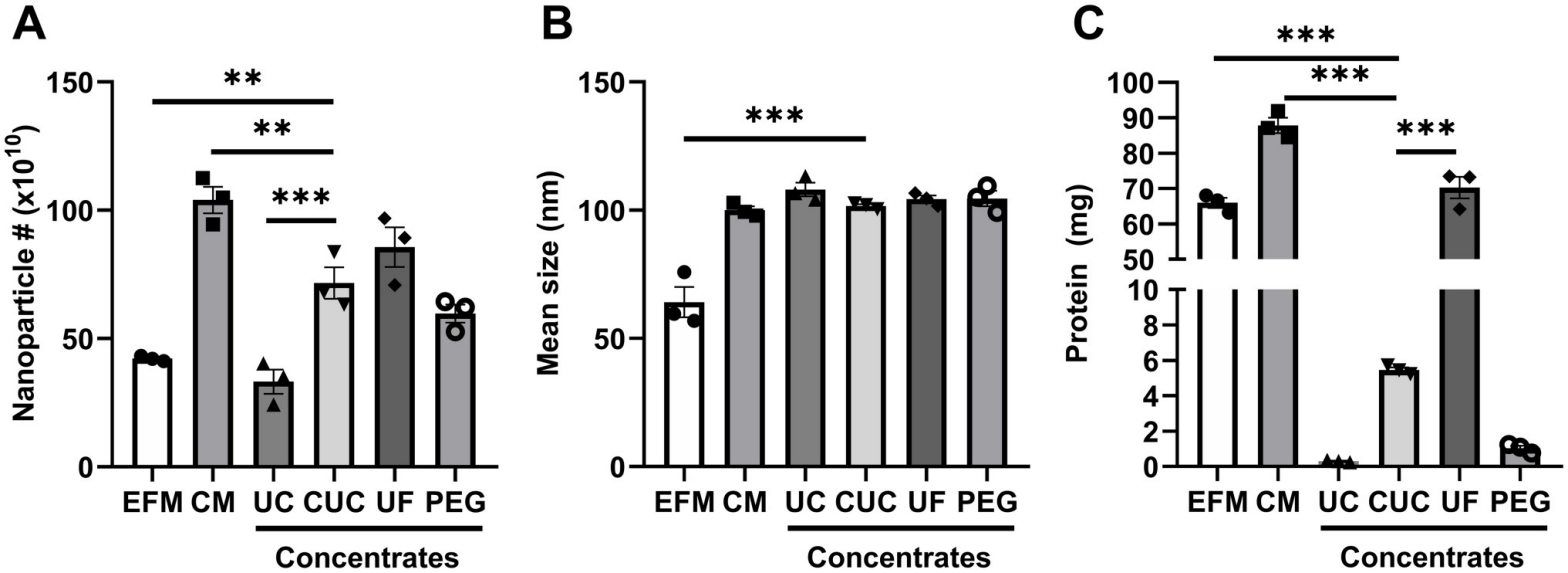
Nanoparticle and protein analysis of concentrates. Using the four different methods, CM was individually concentrated into 1 mL suspensions, which were then mixed with 2 mL 60% iodixanol. NTA served to determine the number (A) and size (B) of nanoparticles in the EFM, the CM, and the 3 mL concentrate from each method. Protein concentration was quantified by Qubit assay (C). For statistical analysis, a one-way ANOVA followed with Dunnett’s multiple comparison test was used, and C-UC served as the control group. Data are expressed as mean ± SEM from three experiments, *P<0.05; **P<0.01; ***P<0.001.

The four methods were assessed by comparing the nanoparticle and protein levels obtained at the completion of the concentration step. Before cell culture, a volume of 36 mL EFM contained a total of 42 x 10^10^ nanoparticles (Fig 2A) with a mean size of 64 nm (Fig 2B) as assessed by Nanoparticle Tracking Analysis (NTA). Following 24 hours of culturing and initial clarification, the number of nanoparticles detected in the CM increased to 104 x 10^10^ nanoparticles and the mean size of particles increased to 100 nm. In addition, the amount of total protein in the CM increased by 25% relative to the EFM (Fig 2C). Concentration by UC resulted in a 30% recovery of nanoparticle (Fig 2A) and a 0.3% retention of total protein seen in the CM (Fig 2C). In contrast, when using C-UC concentration, 70% of all nanoparticles were recovered from the CM along with 6% of total protein. Although UF concentration recovered 82% of nanoparticles from the CM (Fig 2A), it also retained 80% of total protein (Fig 2C). Lastly, concentration by PEG recovered 57% of nanoparticles (Fig 2A) while preserving approximately 1% of total protein (Fig 2C).

### Nanoparticle and protein distribution in DGUC fractions

We next sought to further purify EVs from the four concentrates through DGUC. We refer to these two-step approaches as UC-DGUC, C-DGUC, UF-DGUC, and PEG-DGUC. The concentrates derived from the four methods were adjusted to a final volume of 3 mL that contained 40% iodixanol. These 3 mL concentrates were placed below a step gradient composed of three densities of iodixanol: 5%, 10%, and 20%. After 18 hours of ultracentrifugation at 100,000 x g, twelve 1 mL fractions were collected sequentially starting from the top of the gradient.

**Fig 3:**
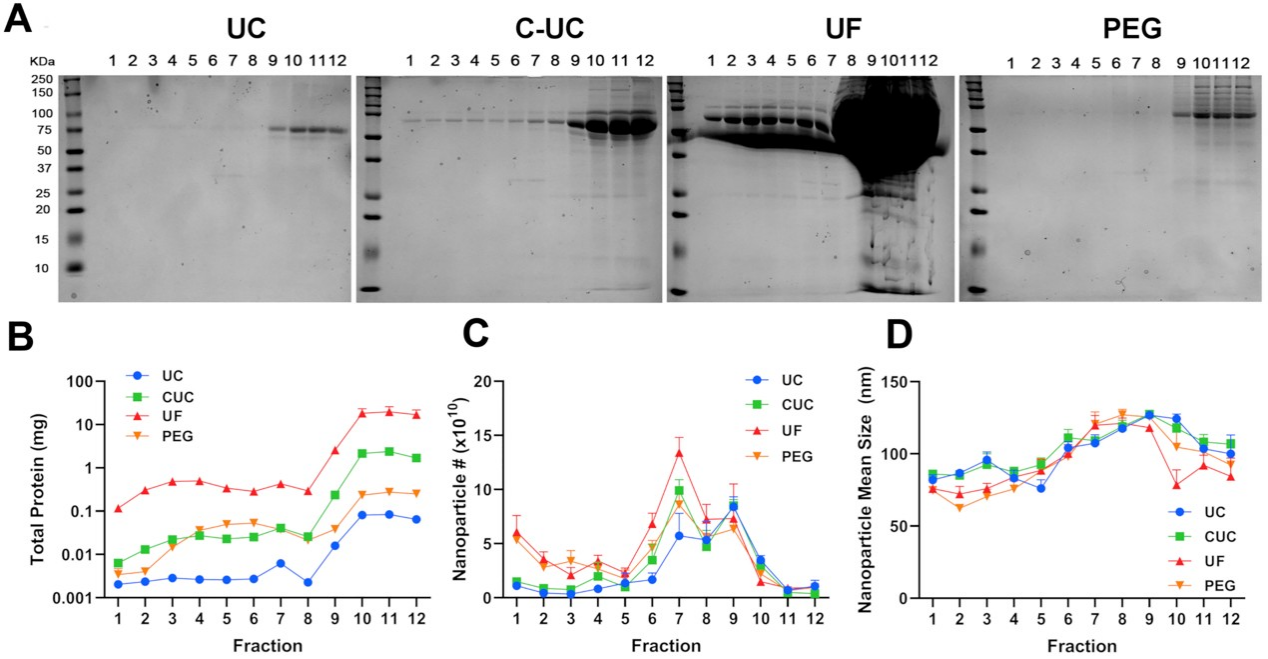
Nanoparticle and protein analysis of DGUC fractions. The 3 mL concentrates from each of the four methods were layered under a discontinuous iodixanol gradient and centrifuged at 100,000 x g. After 18 hours, twelve 1-mL fractions were collected starting from the top. SDS-PAGE served to resolve a 90 μL sample of each fraction for Coomassie staining (A). Protein concentration of each fraction was quantified by Qubit (B). NTA served to analyze the count (C) and mean size (D) of all nanoparticles in each fraction. Data are plotted from three independent experiments as mean ± SEM.

Qubit and Coomassie brilliant blue stain were performed to visualize the protein distribution within the gradient. The majority of the protein was distributed in fractions 9–12 regardless of the concentration method (Fig 3A and 3B). Residual amounts of protein were detected in the first 8 fractions of the UC-DGUC and PEG-DGUC fractions, with C-DGUC having slightly higher levels of protein in these fractions (Fig 3A). Strikingly, the amount of protein found within each fraction of UF-DGUC far exceeded that observed in the other three methods (Fig 3A and 3B).

We next quantified the number of nanoparticles by NTA from each fraction across the four concentration methods. Nanoparticles were present in most fractions with a peak number of nanoparticles in fractions 7 and 9 regardless of the concentration method (Fig 3C). NTA also revealed that fractions 1–5 contained smaller nanoparticles, 82 ± 4 nm, as compared to the later fractions, 6–12 (110 ± 4 nm) (Fig 3D).

### EV markers are similarly distributed amongst the density gradient fractions from the four concentration methods

**Fig 4:**
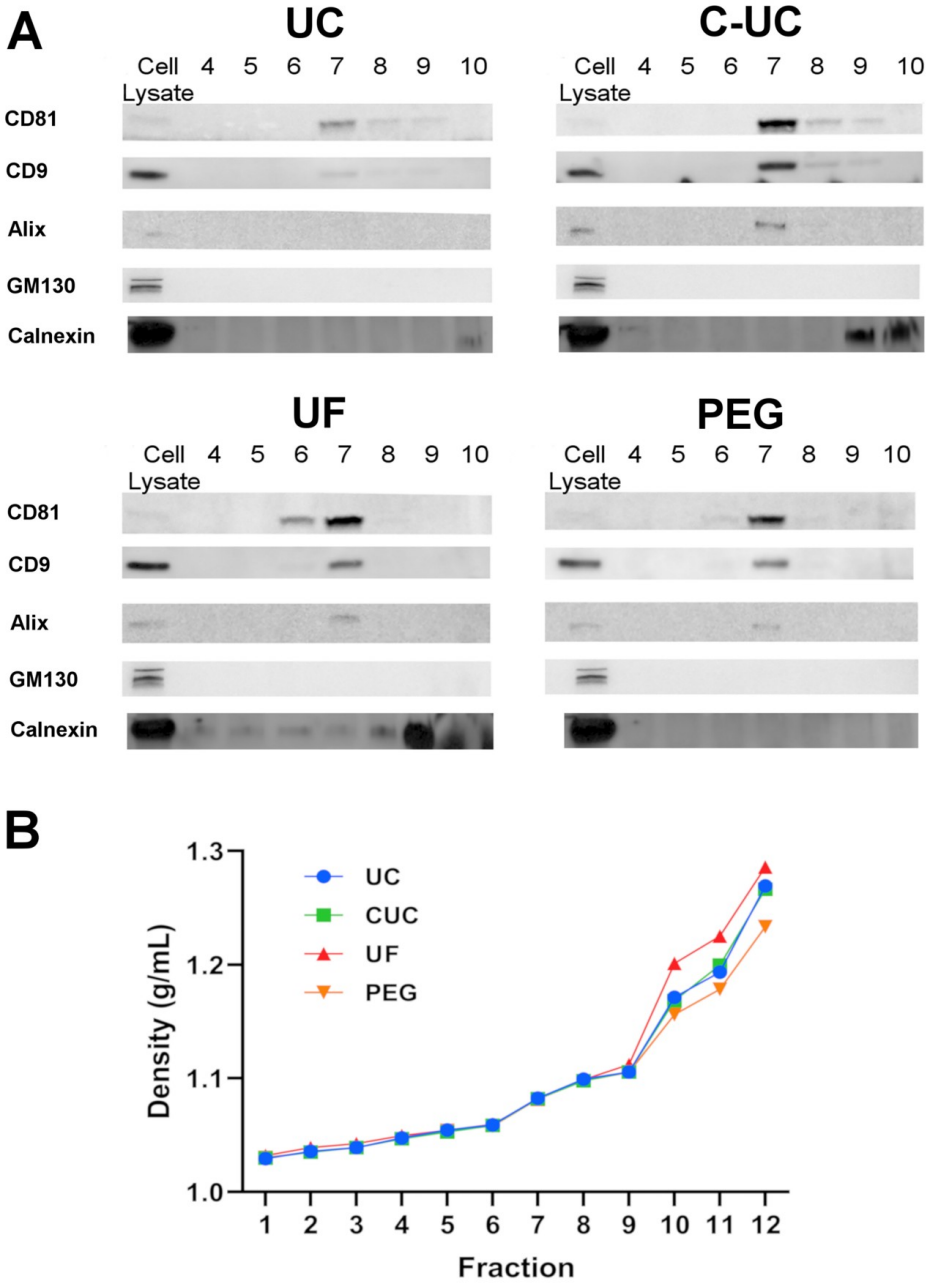
Western blot analysis of DGUC fractions. A 37.5 μL sample was taken from each fraction and resolved by SDS-PAGE and probed for EV markers ALIX, CD81, and CD9. Golgi-derived protein GM130 and endoplasmic reticulum-derived marker Calnexin were used as negative controls (A). The density of each fraction was measured by a refractometer (B). Data are plotted from three independent experiments as mean ± SEM.

Western blots served to confirm the presence of EVs by detecting proteins enriched in EVs including ALIX and the tetraspanins CD9 and CD81. An equal volume of sample from fractions 4 to 10 was used for immunoblotting. The three markers were primarily observed in fraction 7 regardless of the method used to concentrate the EVs. There was minor reactivity in the adjacent fractions, 6 and 8 (Fig 4A). In addition to determining the presence of EV markers, we also assessed the potential co-isolation of vesicles deriving from cellular organelles. As shown in Fig 4A, all fractions tested from the four methods were devoid of GM130, a marker that would indicate vesicles of Golgi origin. Fraction 7 from UC-DGUC, C-DGUC, and PEG-DGUC were also devoid of Calnexin, a marker of vesicles of endoplasmic reticulum (ER) origins. Fraction 7 of UF-DGUC contained a reactivity for calnexin, indicating a co-isolation of ER-derived vesicles.

Next, we sought to determine the density of the isolated fractions. As shown in Fig 4B, the densities of fractions 6 to 8 ranged between 1.07–1.10 g/mL, which agrees with the buoyant density of EVs in iodixanol gradients reported in several prior studies [20, 31–33]. Interestingly, we observed that increased levels of protein might alter the density of later fractions (9 to 12).

### Nanoparticle, protein, and RNA yields of fraction 7

**Fig 5:**
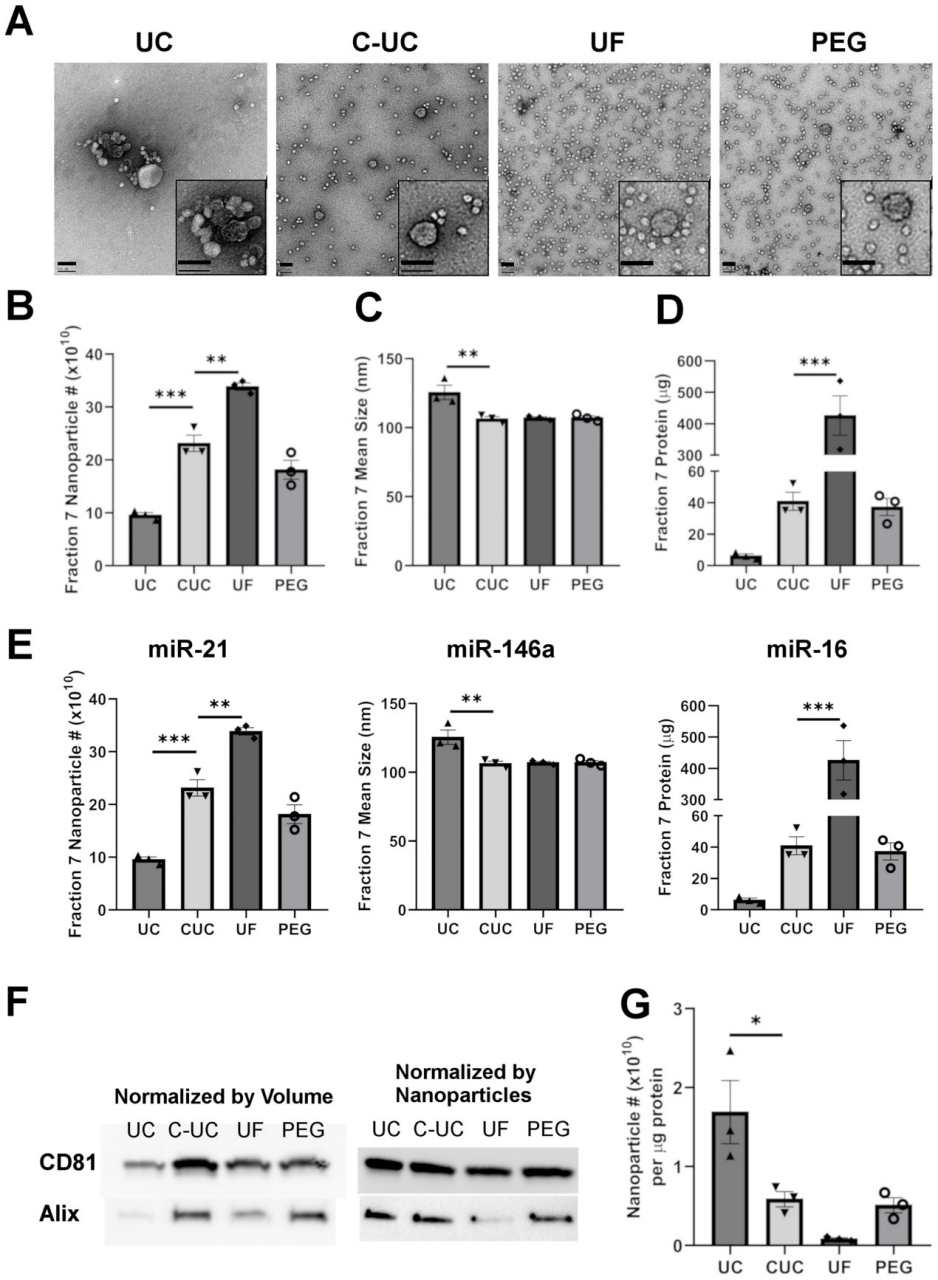
Nanoparticle, protein and RNA analysis of the EV containing fraction. Electron Microscopy of EVs from fraction 7 isolated using different methods with both scale bars representing 100nm (A). Nanoparticles in fraction 7 isolated using different methods were enumerated (B) and sized (C) by NTA. Protein mass was quantified by Qubit assay (D). An equal volume (200 μL) was taken from fraction 7 for miRNA analysis. Levels of microRNAs miR-21, miR-146a and miR-16 were measured relative to the synthetic spike-in UniSp2 by qPCR (E). An equal volume (37.5 μL) and number (3 x10^9^ nanoparticles) from fraction 7 of all four methods were taken and assessed for CD81 and ALIX by western blot. Representative blot images are shown (F). The ratio of nanoparticles count to μg protein was plotted as a relative measurement of purity (G). For statistical analysis, a 1-way ANOVA followed with Dunnett’s multiple comparison test was used, C-UC served as the control group. Data are expressed as mean ± SEM from three experiments, *P<0.05; **P<0.01; ***P<0.001.

Because western blots for EV markers consistently revealed that EVs resided primarily in fraction 7 of the density gradient regardless of the method used to concentrate the CM, this fraction from each method was used for subsequent analyses. To this end, we first confirmed the presence of EVs in fraction 7 by Electron Microscopy (EM) (Fig 5A). The micrographs obtained by EM revealed vesicles containing lipid bilayers with an approximate size of 100 nm. Interestingly, fraction 7 of UC-DGUC showed evidence of EV aggregates in agreement with a prior report [24]. Furthermore, the EM-assessment of fraction 7 from all four methods revealed smaller structures of less than 50 nm that have recently been described as small vesicles and amorphous protein aggregates [34–36].

Concerning nanoparticle recovery, the enumeration as determined by NTA and Qubit revealed that C-DGUC recovered two-fold more particles (Fig 5B) and six-fold more total protein (Fig 5D) compared to UC-DGUC. Although fraction 7 from UF-DGUC revealed a higher yield of nanoparticles relative to that obtained by the other three methods, it retained over tenfold more protein compared to fraction 7 isolated by the C-DGUC method. Finally, fraction 7 of PEG-DGUC recovered slightly fewer nanoparticles and proteins compared to C-DGUC. Interestingly, the mean size of nanoparticles from fraction 7 of UC-DGUC was larger (125nm) than those isolated by the other three concentration methods (107nm), as well as those detected in the CM (100nm) (Figs 5C and 2B). One reason for the large EV sizes could derive from the aggregation of EVs occurring in conventional UC [37].

We next examined the miRNA recovery efficiency by quantifying select miRNAs extracted from an equal volume of fraction 7. We opted to choose miR-21, miR-16, and miR-146a to assess miRNA yield from our prior study of J774A.1 macrophage-derived EVs that identified their presence. The level of miRNAs was normalized to the synthetic spike-in UniSp2. In agreement with NTA and protein assays, fraction 7 from UF-DGUC contained significantly more levels of miR-21 and miR-146a compared to the three other methods, correlating with its increased nanoparticle number and increased protein level. In contrast, fraction 7 from UC-DGUC contained the least amounts of miR-21 and miR-146a, corresponding with its reduced number of nanoparticles and protein recovered by this approach. However, fraction 7 from C-DGUC and PEG-DGUC contained similar levels of miR-21 and miR-146a (Fig 5E).

We sought to further assess the increase in EV yields isolated by C-DGUC through western blotting. The amount of sample from fraction 7 loaded was either normalized to nanoparticle number or total volume. Despite having the greatest nanoparticle count and protein yield, when normalized by volume, fraction 7 of UF-DGUC did not show the most reliable signals for the EV markers ALIX and CD81 (Fig 5F), suggesting a co-isolation of contaminating microparticles. Instead, fraction 7 of C-DGUC showed an increased level of CD81 reactivity compared to all three methods. In addition, the signal intensity for ALIX was similar between C-DGUC and PEG-DGUC. In contrast, the markers CD81 and ALIX, of fraction 7 of UC-DGUC showed the lowest levels, corresponding to its low nanoparticle count and protein recovery. Next, an equal number of nanoparticles (3 x 10^9^) was taken from fraction 7 of each method and probed for the presence of CD81 and ALIX. When examined in this manner, a similar reactivity for CD81 was observed across all four methods. Detection of ALIX, on the other hand, revealed low reactivity in fraction 7 of UF-DGUC while the other three methods displayed similar reactivity (Fig 5F).

Lastly, we sought to assess the purity of fraction 7 by evaluating the nanoparticle count per μg protein ratio as previously proposed [38]. When examined in this manner, UC-DGUC offered the purest preparation amongst the four methods while UF-DGUC offered the least pure preparation. The C-DGUC and PEG-DGUC approaches offered a similar purity with both being superior over UF-DGUC.

## Discussion

Ever since their early report, including that by Rose Johnstone and colleagues [39], investigators have increasingly sought to understand the biological properties of EVs. Seminal findings by Valadi et al. [2] demonstrating the intercellular transfer of RNA by EVs, specifically exosomes, opened a new avenue of research centered on these tiny cellular particles in diverse physiological systems [4, 5]. However, the small size of many EVs including exosomes, ranging from 50 to 150 nm has imposed numerous technical hurdles for their isolation and study. The most popular method remains the one set up by Thery et al. [29] that makes use of ultracentrifugation to concentrate EVs and other microparticles in a pellet that is then resuspended for downstream applications. Because UC is limited in scale, cumbersome, and requires costly equipment numerous other traditional protein and virus concentration methods have been adopted for concentrating these vesicles. Ultrafiltration is a well-established method making use of membrane separation based on molecular size. When using this method, EVs and molecules more massive than the membrane pores are retained and concentrated [23]. The use of polyethylene glycol, which long served as a fractional precipitating agent for virus [40] and lipoprotein sedimentation from plasma [41], has also gained popularity due to its rapid and simple-to-use nature. Because polyethylene glycol displays high solubility in water, EVs and macromolecules are sterically excluded from the solvent and can be concentrated by low-speed centrifugation [42].

As highlighted in the Minimal Information for Studies of Extracellular Vesicles 2018 (MISEV2018), several criteria should be considered when selecting methods for EV purification: (a) yield defined by maximizing the recovery of EVs while concentrating them from a large volume; (b) specificity that includes minimizing the retention of protein and non-vesicular nanoparticles in the concentrates; (c) biological integrity: which is achieved when preserving functional properties of these EVs [43]. In UC, nanoparticles sediment at rates determined by their sedimentation coefficients, which largely depend on their size. This property of centrifugation explains in part the cross-contamination that occurs when using UC in the absence of a second purification step. UF, on the other hand, excludes particles based on their molecular size, which is defined by the molecular weight cut-off of the semipermeable membrane. Since there is a significant size overlap between EVs, other vesicles, as well as large protein, this method leads to the retention of all these entities [44]. PEG precipitation is similarly incapable of discriminating EVs from non-EV nanoparticles and protein.

A key finding of our study is that the choice of the concentration approach has a substantial impact on the yield and purity of EVs. In agreement with multiple reports [27, 45, 46], our results show that UC resulted in a marked loss of nanoparticles during the concentration step. This approach yielded a modest 30% recovery of nanoparticles from the CM. Some recent studies have reported evidence that the physical integrity and biological function of EVs including exosomes isolated by ultracentrifugation may be compromised due to EV aggregation [24]. Indeed, our Transmission Election Microscopy data (Fig 5A) support this possibility.

Furthermore, our data show that the use of a liquid cushion composed of 60% iodixanol as in the C-UC method substantially improves the recovery rate reaching 70% of all nanoparticles detected in the CM. There are at least two reasons that could explain why C-UC provides a robust recovery of most nanoparticles including from the CM. First, the approach avoids pellet formation and thereby likely prevents EV aggregation, preserving their physical integrity and stability. Second, this approach collects 1 mL of solution above the high-density cushion which probably traps most EVs due to its high viscosity. In addition to greater EV yields, C-UC also offers an improvement in reproducibility. Specifically, the cushion layer avoids the formation of an unstable EV pellet that has recently been noted to be easily lost [47], thus reducing user-dependent recovery rates and thereby enhancing the consistency of the isolation.

Our study also revealed that the method of concentration determines the level of protein contaminants recovered in the EV isolate after DGUC. The concentration of conditioned media using ultrafiltration devices results in similar EV recovery efficiency as the C-UC method. However, the UF concentrate contained substantially more protein contaminants. Importantly, such protein contaminants cannot be entirely removed following the subsequent DGUC step. This results in a tenfold increase of protein contaminants in UF EV isolates compared to those isolated from C-UC. This finding suggests that caution should be exercised when UF-like methods such as tangential flow filtration (TFF) are employed to concentrate conditioned media. TFF may offer a scalable option to work with large volumes of fluid which is difficult to achieve by ultracentrifugation-based methods. However, consistent with our finding, TFF has been reported to retain a significant amount of protein contaminants [48, 49]. For that reason, a second purification step such as size exclusion chromatography is recommended to purify further TFF concentrates [49]. Furthermore, the efficiency of EV isolation using UF based-concentration methods can be improved by utilizing a different centrifugal filter. Specifically, the Amicon Ultra-2 10k has been shown to outperform its 100kDa MWCO counterpart [44] such as the one that was used in this study.

Our study also reveals another option for large-scale isolation of EVs from conditioned media by employing precipitation using PEG coupled with a DGUC approach. The use of PEG allows EVs from a large volume of media to be precipitated and isolated using a low-speed, more accessible centrifugation system. Our data suggest a similar performance between PEG and C-UC concerning nanoparticle, protein, and miRNA recovery. Therefore, PEG offers an option to remedy the limitations of ultracentrifugation for the initial concentration step, as recently highlighted by Ludwig et al. [47]

The ratio of particles to protein has been proposed to measure EV purity [38]. Based on this parameter, the EV-containing fraction generated by UF-DGUC is by far the most contaminated relative to the other three methods. UC-DGUC offered the purest preparation while C-DGUC and PEG-DGUC shared a similar purity index. However, the ratio of particle to protein does not fully account for other potential contaminants such as fragmented DNA, which might be co-isolated with certain approaches. PEG, for example, has been employed to isolate DNA and lipoproteins [41, 50–52]. Nonetheless, it is beyond the scope of this study to identify and characterize contaminants generated by all four methods.

In summary, success in EV research relies on the ability to isolate pure and biologically active EVs from cell culture media and biofluids. Our data demonstrate that concentration by C-UC provides significant improvements in exosome recovery compared to UC. When performed in combination with density gradient ultracentrifugation, C-DGUC may significantly enhance the reproducibility of downstream characterization and functional studies of EVs isolated from cell culture media. Although UC-DGUC offers purer isolation regarding protein-nanoparticle ratio, the introduction of an iodixanol cushion in C-DGUC prevents the pelleting and possible aggregation of EVs thereby protecting their integrity. Furthermore, it is possible that a higher level of purity could be achieved by modifying our current C-DGUC method. In this study, we chose to take 1mL above the iodixanol cushion that may include undesired protein contaminants from the conditioned media. Therefore, reducing the volume taken may enhance the purity of the isolation of EVs without substantial losses. Furthermore, ongoing studies including those from our lab are exploring the application of C-DGUC in the separation of EVs and exosomes from more complex biofluids such as blood plasma.
