# Correction: Cushioned-Density Gradient Ultracentrifugation (C-DGUC) improves the isolation efficiency of extracellular vesicles

**DOI:** 10.1371/journal.pone.0236914

**Published:** 2020-07-23

**Authors:** Phat Duong, Allen Chung, Laura Bouchareychas, Robert L. Raffai

In Fig 5, panel E erroneously displays the histograms shown in panels B, C, and D. Please see the correct Fig 5 here.

**Fig 5:**
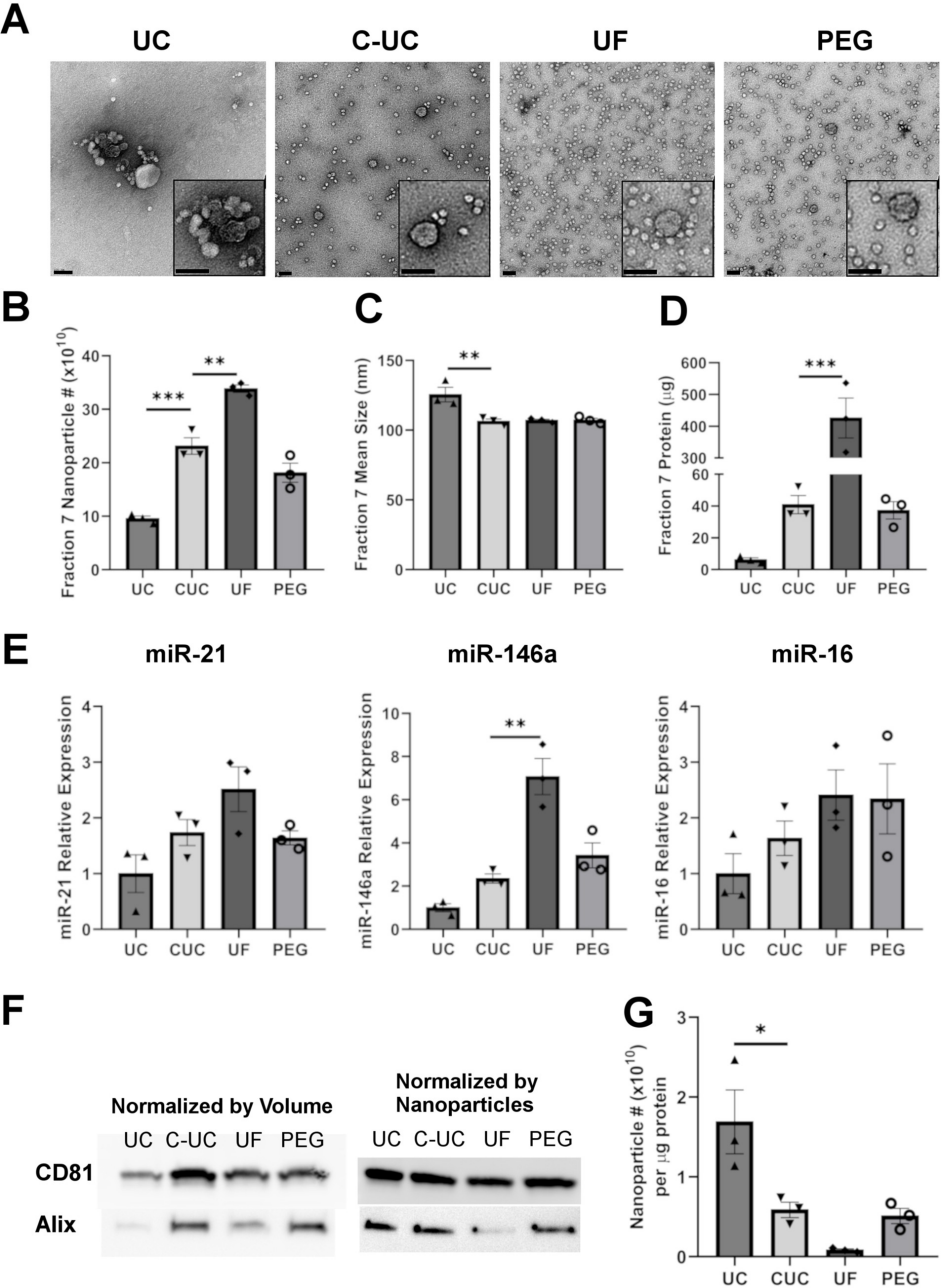
Nanoparticle, protein and RNA analysis of the EV containing fraction. Electron Microscopy of EVs from fraction 7 isolated using different methods with both scale bars representing 100nm (A). Nanoparticles in fraction 7 isolated using different methods were enumerated (B) and sized (C) by NTA. Protein mass was quantified by Qubit assay (D). An equal volume (200 μL) was taken from fraction 7 for miRNA analysis. Levels of microRNAs miR-21, miR-146a and miR-16 were measured relative to the synthetic spike-in UniSp2 by qPCR (E). An equal volume (37.5 μL) and number (3 x10^9^ nanoparticles) from fraction 7 of all four methods were taken and assessed for CD81 and ALIX by western blot. Representative blot images are shown (F). The ratio of nanoparticles count to μg protein was plotted as a relative measurement of purity (G). For statistical analysis, a 1-way ANOVA followed with Dunnett’s multiple comparison test was used, C-UC served as the control group. Data are expressed as mean ± SEM from three experiments, *P<0.05; **P<0.01; ***P<0.001.
